# The reversal of recurrence hazard rate between ER positive and negative breast cancer patients with axillary lymph node dissection (pathological stage I-III) 3 years after surgery

**DOI:** 10.1186/1471-2407-8-323

**Published:** 2008-11-07

**Authors:** Takayoshi Kiba, Takashi Inamoto, Tsutomu Nishimura, Masaya Ueno, Kazuhiro Yanagihara, Satoshi Teramukai, Hironori Kato, Masakazu Toi, Masanori Fukushima

**Affiliations:** 1 Outpatient Oncology Unit, Kyoto University Hospital, Kyoto, Japan; 2 Translational Research Informatics Center, Kobe, Japan; 3 Department of Breast Surgery, Kitano Hospital, The Tazuke Kofukai Medical Research Institute, Osaka, Japan; 4 Translational Research Center, Kyoto University Hospital, Kyoto, Japan; 5 Department of Breast Surgery, Graduate School of Medicine, Kyoto University, Kyoto, Japan

## Abstract

**Backgrounds:**

Prognostic factors are defined as biological or clinical measurement associated with overall survival and/or disease-free survival. Previous studies have shown that patients with estrogen receptor (ER) positive cancers have a better prognosis than patients whose cancers do not have these receptors.

**Methods:**

This study investigated the assessment of variables in defining prognosis of 742 breast cancer women with pathological stage (pTNM) I-III diagnosed between 1980 and 2005 at the Kyoto University Hospital in Japan, by age, clinical stage (cTNM), pTNM, the numbers of positive lymph nodes (pN), and ER status.

**Results:**

Multivariate analysis demonstrated that pTNM and ER status were the independent prognostic factors for overall survival, and that pTNM and pN were the independent prognostic factors for disease-free survival. For the 0- to 2-year interval, the hazard of recurrence was higher for the ER-negative patients than the ER-positive patients, and beyond 3 years the hazard was higher for ER-positive patients.

**Conclusion:**

The present study confirmed the previous reports which showed favorable prognosis of the patients with lesser pTNM or positive ER status. A reversal of recurrence hazard rate between ER positive and negative breast cancer patients beyond 3 years after operation was detected. The fact may indicate the importance of long term adjuvant hormone therapy for ER positive cancer patients.

## Background

A prognostic factor is defined as a biological or clinical measurement that is associated with overall survival and/or disease-free survival [1]. The knowledge of prognosis forms an integral part of the decision-making process in medicine [2]. Moreover, prognostic factors are important in the treatment of cancer to help identify subgroups of patients who may need more aggressive approach to therapy [3]. Further, prognostic factors also play a critical role in designing clinical trial as stratification and allocation factors [4]. Prognostic factors, i.e., those that predict the risk of recurrence or death from breast cancer, include stage, number of positive axillary nodes, tumor size, lymphatic and vascular invasion, the estrogen-receptor (ER) and progesterone-receptor (PR) positivity, and HER2/neu gene amplification [3,5]. We previously reported that the recent advance of the survival rates in breast cancer patients may be due to the rational development of treatment [6]. In order to assess the independent value of variables in defining prognosis, in the present study, we have investigated the survival of 742 breast cancer patients with pathological stage (pTNM) I-III, by the age, clinical stage (cTNM), pTNM, the numbers of positive lymph nodes (pN) and ER status.

## Methods

### Patients

742 female beast cancer patients aged between 21 and 80 with stage I-III of pTNM were selected from the patients treated at Kyoto University Hospital in Japan from 1980 to 2005. Based on the section 2 in chapter 1 of Japanese ethical guidelines for epidemiological research http://www.niph.go.jp/english2/english ver/ethical-gl/guidelines.htm, this study was exempt from ethical approval under Japanese law and guidelines. Moreover, all treatments for breast cancer were undertaken with informed consent and consents were also taken to confirm cancer diagnosis. These patients underwent surgery with axillary lymph node dissection. The operation methods were classified into three groups: breast conserving surgery, modified radical mastectomy, and standard radical mastectomy. All the patients with breast conserving surgery received radiation therapy. Staging of cTNM and pTNM was evaluated according to UICC stage [7]. Number of lymph node metastasis and ER status of the primary tumors were analyzed by staff members of the Department of Pathology at Kyoto University Hospital. Using immunohistochemistry on the whole series of tumors, they assessed estrogen receptor (ER) status in a standardized way. In our institute, the pathologists routinelyhave examined the ER status of tumors by using the immunohistochemistry since the 1980s. The contents of treatments for breast cancer patients were previously described [6]. According to the years of surgery the patients were grouped into two cohorts: period I (1980–1989) and period II (1990–2005). In period I, modified radical mastectomy with lymph node dissection was included. In this period, breast-conserving surgery was not performed, because it was not recognized to be the prevailing method in Japan. In period II, breast-conserving surgery was the treatment of choice for women with relatively small breast cancers during this past decade in Japan. In our institute, all patients with breast-conversing surgery received radiation therapy. In the treatment stage I, II, IIIA, and operable stage IIIC breast cancer, breast-conserving surgery or modified radical mastectomy with lymph node dissection and with or without breast reconstruction surgery was included. In the treatment of stage IIIB and inoperable stage IIIC breast cancer, systemic chemotherapy, or systemic chemotherapy followed by surgery, with lymph node dissection followed by radiation therapy were included. If necessary, additional systemic therapy such as chemotherapy, hormone therapy, or both were given. Moreover, if necessary, adjuvant therapy such as systemic chemotherapy (*per os *only) with or without hormone therapy (tamoxifen or tremifene) was included. The patients received adjuvant chemotherapy with LH-RH agonist after 2001, cyclophosphamide, epirubicin and 5-fluorouracil (CEF) or Cyclophosphamide, methotrexate and 5-fluouracil (CMF) regimen after 2002, and rational developers such as taxane, trastuzumab, or aromatase inhibitor therapy after 2004.

### Statistical analysis

Disease-free survival was defined from the operation day to the identification date of recurrence of cancer or death from any cause, and overall survival was defined from the operation day to death from any cause. Survival curves were estimated with the Kaplan-Meier method. To identify prognostic factors independently associated with the overall survival or disease-free survival and to estimate the hazard ratios, the Cox proportional hazard model was applied. Two-sided *p *<*0.05 *was regarded as statistically significant. The statistical analysis was conducted with SPSS version 11.0 statistical software.

## Results

### Patient Characteristics

Patient characteristics are summarized in Table 1. The medianfollow-uptime of the investigated period in this study was as same as the median follow-up time forsurvivingpatients (5.7 years).

**Table 1:** Patient characteristics (n = 742)

	number	%
Gender		
female	742	100
male	0	0
Age		
<35 (21–34)	35	4.7
35–54	337	45.4
≥ 55 (55–91)	370	49.9
cTNM stage		
Stage I	197	26.6
Stage II	452	60.9
Stage III	93	12.5
pTNM stage		
Stage I	189	25.5
Stage II	397	53.5
Stage III	156	21.0
pN		
pN0	422	56.9
pN1	189	25.5
pN2	88	11.9
pN3	43	5.8
ER status		
negative	290	39.1
positive	452	60.9
Breast surgery		
Breast conserving surgery	305	41.1
Modified radical mastectomy	429	57.8
Standard radical mastectomy	8	1.1

### 10-year overall survival

**Figure 1:**
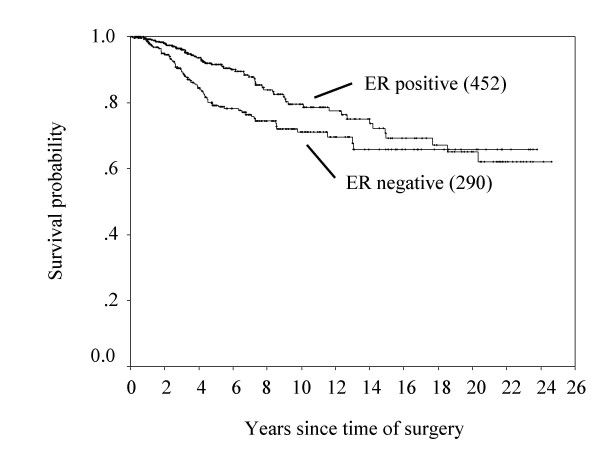
**Overall survival curves in ER-positive and ER-negative patients.** (Number) = number of patients. *p = 0.012*.

The 10-year overall survival rates classified by age, cTNM, pTNM, pN, ER status and types of breast surgery are shown in Table 2. Figure 1 shows the overall survival curves in ER-positive and ER-negative patients.

**Table 2:** The 10-year overall survival rates and univariate Cox regression analysis

Factors	overall survival rates		Hazard ratio		Log-rank test *p*-value
				
	10-year (%)	95% CI^a^		95% CI ^a^	
Age					
< 35	69.6	57.7–81.5	1.00	-	0.30
35–54	78.1	75.2–81.0	0.69	0.30–1.59	
≥ 55	73.4	69.9–77.0	0.90	0.39–2.08	
cTNM					
Stage I	85.7	81.3–90.0	1.00	-	<0.001
Stage II	75.8	73.1–78.5	2.32	1.31–4.09	
Stage III	54.5	46.9–62.0	4.85	2.55–9.22	
pTNM					
Stage I	89.4	85.8–93.1	1.00	-	<0.001
Stage II	81.7	79.1–84.4	2.10	1.10–4.03	
Stage III	46.4	40.8–51.9	7.77	4.08–14.81	
pN					
pN0	86.7	84.2–89.1	1.00	-	<0.001
pN1	76.4	72.1–80.7	1.74	1.08–2.82	
pN2	46.6	39.7–53.4	5.25	3.34–8.27	
pN3	38.2	26.9–49.5	5.34	3.01–9.47	
ER status					
Negative	71.0	67.7–74.3	1.00	-	0.012
Positive	79.5	76.5–82.5	0.63	0.44–0.91	
Breast surgery					
Breast conserving surgery	76.1	71.2–81.0	1.00	-	0.093
Modified radical mastectomy	76.2	73.7–78.7	1.31	0.84–2.04	
Standard radical mastectomy	19.1	2.29–35.8	4.09	1.57–10.64	

^a ^CI: Confidence interval.

### 10-year disease-free survival

**Figure 2:**
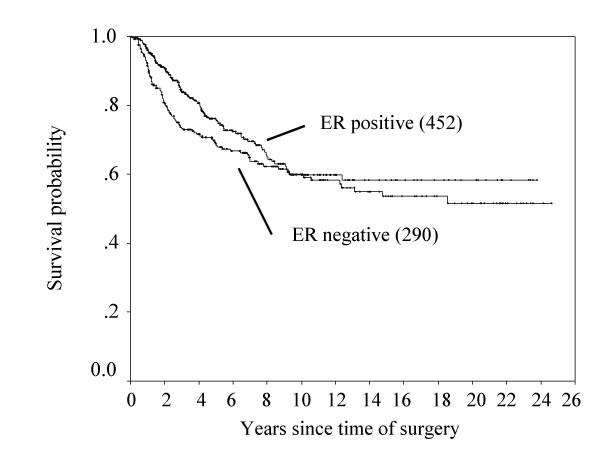
**Disease-free survival curves in ER-positive and ER-negative patients.** (Number) = number of patients. *p = 0.18*.

The 10-year disease-free survival rates classified by age, cTNM, pTNM, pN, ER status and types of breast surgery are shown in Table 3. The approximate 10-year disease-free survival between ER positive and negative patients was reversed (Figure 2). According to age, cTNM, pTNM and pN, the reversal of disease-free survival was not detected in the present study (Table 3).

**Table 3:** The 10-year disease-free survival rates and univariate Cox regression analysis

Factors	disease-free survival rates		Hazard ratio		Log-rank test *p*-value
				
	10-year (%)	95% CI ^a^		95% CI ^a^	
Age					
< 35	47.0	33.3–60.7	1.00	-	0.49
35–54	59.0	55.7–62.4	0.91	0.50–1.64	
≥ 55	63.1	59.4–66.7	0.90	0.50–1.63	
cTNM					
Stage I	72.4	67.3–77.6	1.00	-	<0.001
Stage II	60.9	58.0–63.9	2.05	1.45–2.91	
Stage III	31.5	24.8–38.2	5.03	3.36–7.52	
pTNM					
Stage I	81.7	77.4–85.9	1.00	-	<0.001
Stage II	67.5	64.4–70.5	2.18	1.47–3.24	
Stage III	17.7	13.1–22.4	7.66	5.13–11.43	
pN					
pN0	76.6	73.8–79.5	1.00	-	<0.001
pN1	56.4	51.6–61.2	1.85	1.36–2.53	
pN2	15.5	10.3–20.7	5.75	4.22–7.83	
pN3	26.8	16.6–37.1	4.88	3.25–7.32	
ER status					
Negative	59.8	56.4–63.2	1.00	-	0.183
Positive	60.0	56.6–63.4	0.83	0.63–1.09	
Breast surgery					
Breast conserving surgery	59.1	54.1–64.0	1.00	-	0.007
Modified radical mastectomy	61.5	58.7–64.3	1.14	0.86–1.52	
Standard radical mastectomy	ND^b^	-	3.34	1.76–6.33	

^a ^CI: Confidence interval,^b ^ND: not determined.

### Estrogen receptor status

**Figure 3:**
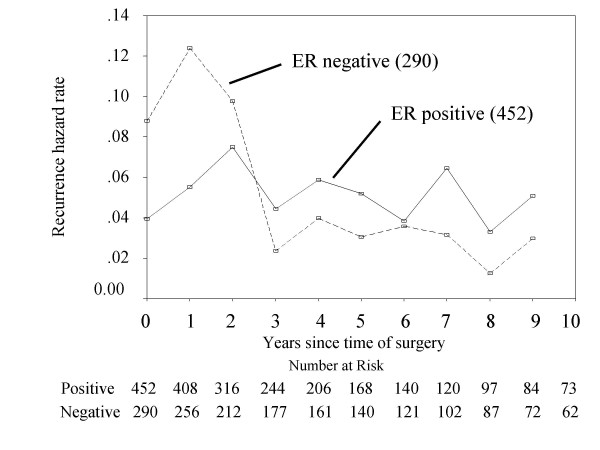
**Annual hazard of recurrence of patients separated by ERstatus.** (Number) = number of patients.

**Figure 4:**
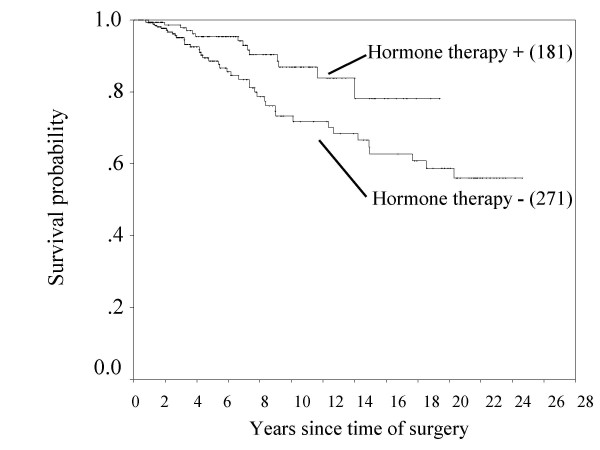
**Overall survival curves in ER-positive patients with and without adjuvant hormone therapy.** (Number) = number of patients. *p = 0.009*.

**Figure 5:**
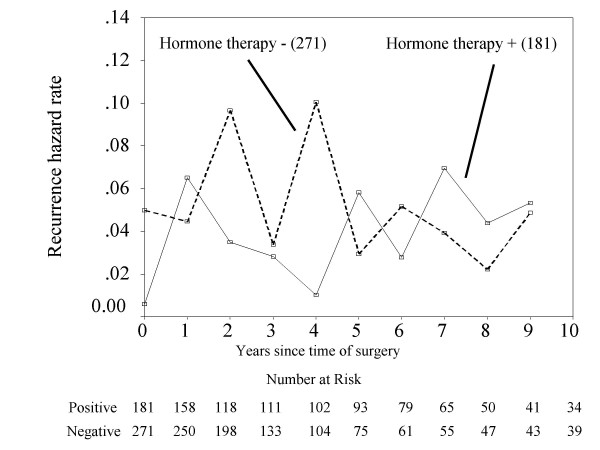
**Annual hazard of recurrence of ER-positive patients separated by adjuvant hormone therapy.** (Number) = number of patients.

Because beyond 10 years hazard had increased statistical errors, we investigated the annual hazard of recurrence until 10 years after operation. For the 0- to 2-year interval, the hazard of recurrence was higher for the ER-negative patients than the ER-positive patients, and beyond 3 years the hazard was higher for ER-positive patients (Figure 3). Figure 4 shows that the overall survival of ER-positive cancer patients was increased by adjuvant hormone therapy (*p = 0.009*). Moreover, among 452 ER-positive cases, at 1 year after surgery, the hazard of recurrence was higher for the patients with adjuvant hormone therapy than the patients without adjuvant hormone therapy, but between 2 and 4 years, the hazard was higher for the patients without adjuvant hormone therapy (Figure 5).

### Prognostic factor analysis

Age (<35; 35–54; ≥ 55), cTNM (stage I-III), pTNM (stage I-III), pN (pN0, pN1, pN2, pN3), ER status (negative, positive, unknown), and types of breast surgery (breast conserving surgery, modified radical mastectomy, radical mastectomy) were analyzed as potential prognostic factors by the Cox proportional hazard model. Both univariate analyses to determine prognostic factors associated with overall survival and disease-free survival that the features with *p *< 0.05 were 5 features: cTNM, pTNM, pN, ER status, and type of surgery (Table 2 &3). The important prognostic factor associated with overall survival determined by multivariate analyses with backward variables selection were 2 features: pTNM and ER status (Table 4). The important prognostic factor associated with disease-free survival determined by multivariate analyses with backward variables selection were two features: pTNM and pN (Table 4).

**Table 4:** Multivariate Cox regression analysis for overall survival and disease-free survival.

Factors	Overall survival		
	
	Hazard ratio	95% CI ^a^	*p*-value
pTNM			
Stage I	1.00	-	
Stage II	2.05	1.07–3.94	0.03
Stage III	8.09	4.24–15.43	<0.001
ER status			
Negative	1.00	-	
Positive	0.57	0.40–0.82	0.002

Factors	Disease-free survival		
	
	Hazard ratio	95% CI ^a^	*p*-value

pTNM			
Stage I	1.00	-	
Stage II	1.87	1.12–3.11	0.017
Stage III	3.72	1.59–8.70	0.002
pN			
pN0	1.00		
pN1	1.49	1.01–2.20	0.044
pN2	2.47	1.14–5.34	0.022
pN3	1.91	0.83–4.39	0.129

^a ^CI: Confidence interval

## Discussion

Tumor staging systems provide information about extent of disease that can be used to guide treatment recommendations and provide estimates of patient prognosis. It is well known that pathological stage is the most significant independent prognostic factor for determining survival in breast cancer [8]. Our study documents the fact that pathological stage is the independent prognostic factor for both overall survival and disease-free survival.

Many studies have shown that women with ER positive cancers have a better prognosis than patients whose cancers do not have this receptor [9,10]. In this study cohort, ER status were the independent prognostic factors for overall survival by the multivariate Cox regression analysis, but ER status did not affect disease-free survival (Table 3 &4). Nomura et al. [11] previously reported that in a retrospective multicenter study to investigate the ER status in primary breast cancer with patient prognosis, 3,118 patients with operable breast cancer (stages I-III) were investigated from ten hospitals in Japan who underwent surgery from October 1972 to December 1982, and that Cox's multivariate analysis showed that overall survival, but not disease-free survival was affected by ER status. They speculated the possibility that this was due to the longer postrelapse survival in patients with ER-positive cancer based on the effectiveness of endocrine treatment. Preceding paper has reported that the patients of positive ER status enjoyed benefits from the recent development of breast cancer treatments [6]. In fact, the present study showed that the overall survival of ER-positive cancer patients was increased by adjuvant hormone therapy (Figure 4).

Hortobagyi et al. [12] previously reported that the disease-free survival in estrogen receptor (ER) positive and/or progesterone receptor (PgR) positive patients was higher than that in ER/PgR negative patients until 5 years after administration of the state-of-the-art adjuvant therapy, however, the disease-free survivals between these groups was reversed after 5 years. Saphner et al. [13] reported that compared with ER negative patients, ER positive patients had lower annual hazard of recurrence until around 3.5 years after surgery, but thereafter higher. In the present study, Figure 3 shows that a positive ER status was associated with a lower hazard of recurrence in the first 2 years after surgery, but a higher hazard of recurrence from years 3 to 10. [14]. Results from the EBCTCG meta-analysis of systemic treatment of early breast cancer by hormone, cytotoxic, or biologic therapy methods in randomized trials involving 144,939 women show a highly significant advantage of 5 years versus 1 to 2 years of tamoxifen with respect to the risk of recurrence [14]. In the present study, in ER-positive cases, between 2 and 4 years after surgery, the hazard of recurrence of patients without adjuvant hormone therapy was higher than the patients with adjuvant hormone therapy (Figure 5). It is noteworthy that this observation emphasizes the importance of adjuvant hormone therapy for ER positive cancer patients beyond 3 years after operation. Moreover, comparing with the 10-year survival rate between ER-positive patients with or without hormone therapy and ER-negative patients (Figure 1 &4), the survival rate between ER-positive patients without hormone therapy and ER-negative patients was similar, but the adjuvant hormone therapy led about 13% survival gains. Therefore, this fact also suggests adjuvant hormone therapy may have more important roles in the treatment. In addition, the disease-free survival at 10 years after surgery between ER positive and negative patients was reversed (Figure 2). This may be related to the fact that the percentage of number of patients who received adjuvant hormone therapy in ER positive patients between 1980 and 1991 (11/84: 13%) was smaller to that between 1991 and 2005 (170/368: 46%), because of reasons including poor understanding of modern treatment for adjuvant chemotherapy, the cost for drugs, and so on. On the other hand, the current recommendation is that adjuvant tamoxifen be discontinued after 5 years in all patients as current standard therapy, because there was a trend toward a worse outcome associated with a longer duration of treatment [15]. Further analyses may be needed to clarify the optimal duration of adjuvant hormone therapy in operated breast cancer patients.

Traditional prognostic factors, i.e., those that predict the risk of recurrence or death from breast cancer, include number of positive axillary nodes [3]. It has been reported that the pN is the most important prognostic factor affecting disease-free survival and overall survival in operable breast cancer patients [2]. However, our study suggested that pN is the independent prognostic factor for disease-free survival, but not for overall survival. The patients with axillary lymph node metastasis have received chemotherapy, hormonal therapy or both. Over the past 20 years, various systemic adjuvant therapies have been studied to improve survival [6]. Therefore, there may be a possibility that the other factors such as these therapies may affect the overall survival more stronger than pN, although further investigations are needed to clarify this matter.

The univariate Cox regression analysis for overall survival and disease-free survival demonstrated that the hazard ratio of patients with breast conserving surgery was lower than that of patients with standard radical mastectomy (Table 2 &3). This fact suggests that breast conserving surgery with radiation therapy may provide not only cosmetic benefit but also better prognosis, although chronological change of breast cancer treatments may affect the survival rates.

In conclusion, the present study presented the data of the long term survival of pathological stage I-III patients with breast cancers at our institution. For the 0- to 2-year interval, the hazard of recurrence was higher for the ER-negative patients than the ER-positive patients, and beyond 3 years the hazard was higher for ER-positive patients. Additionally, disease free survival 10 years after operation was reversed between ER-positive and negative patients. Therefore, the fact may indicate the importance of long term adjuvant hormone therapy for ER positive cancer patients.

## Abbreviations

cTNM: clinical stage; ER: estrogen receptor; pTNM: pathological stage; pN: positive lymph nodes.

## Competing interests

The authors declare that they have no competing interests.

## Authors' contributions

TI and MF designed this study. MU, KY, HK, TI collected and assembled the data. TI organized the data. TK, TN, ST, TI and MF contributed to the statistical analyses and interpretations. TK, TI, MT and MF contributed to writing and finalizing of the manuscript. All authors read and approved the final manuscript.

## Pre-publication history

The pre-publication history for this paper can be accessed here:

http://www.biomedcentral.com/1471-2407/8/323/prepub
